# Non-Contrast Computed Tomography-Based Triage and Notification for Large Vessel Occlusion Stroke: A Before and After Study Utilizing Artificial Intelligence on Treatment Times and Outcomes

**DOI:** 10.3390/jcm14041281

**Published:** 2025-02-15

**Authors:** Yong Su Lim, Eunji Kim, Woo Sung Choi, Hyuk Jun Yang, Jong Youn Moon, Jae Ho Jang, Jinseong Cho, Jeayeon Choi, Jae-Hyug Woo

**Affiliations:** 1 Department of Emergency Medicine, Gil Medical Center, Gachon University College of Medicine, Incheon 21565, Republic of Korea; yongem@gilhospital.com (Y.S.L.); yanghj@gilhospital.com (H.J.Y.); jhjang@gilhospital.com (J.H.J.); chjy14664@gilhospital.com (J.C.); emmetalkiller@gilhospital.com (J.-H.W.); 2 Department of Preventive Medicine, Gachon University College of Medicine, Incheon 21565, Republic of Korea; eunjikim@gachon.ac.kr (E.K.); moon@gilhospital.com (J.Y.M.); 3 Artificial Intelligence and Big-Data Convergence Center, Gil Medical Center, Incheon 21565, Republic of Korea

**Keywords:** large vessel occlusion (LVO) stroke, non-contrast CT (NCCT), artificial intelligence (AI), Heuron ELVO, endovascular thrombectomy (EVT), hyperdense arterial sign (HAS), Alberta Stroke Program Early Computed Tomography Score (ASPECTS), stroke workflow, National Institutes of Health Stroke Scale (NIHSS)

## Abstract

**Background/Objectives**: The clinical impact of automated large vessel occlusion (LVO) detection tools using non-contrast CT (NCCT) is still unknown. We evaluated whether the implementation of Heuron ELVO, an artificial intelligence (AI)-driven software for triage and notification of LVO stroke using NCCT, can reduce treatment times and improve clinical outcomes in a real-world setting. **Methods**: We compared patients with LVO stroke before (pre-AI cohort, 84 patients) and after (post-AI cohort, 48 patients) the implementation of Heuron ELVO at a comprehensive stroke center. Primary outcomes included time-to-treatment initiation, including door-to-IV tPA and door-to-endovascular thrombectomy (EVT) times. Secondary outcomes measured changes in the National Institute of Health Stroke Scale (NIHSS) and modified Rankin Scale (mRS) scores. Statistical analyses involved multiple linear regression to adjust for confounders. **Results**: The implementation of Heuron ELVO significantly reduced the door-to-EVT time (30.2 min, 95% CI, −56. to −4.3), CT-to-neurologist examination time (16.4 min, 95% CI, −27.6 to −5.3), and CT-to-EVT time (29.4 min, 95% CI, −53.6 to −5.0). There was no statistical difference in the door-to-IV tPA time (8.9 min). The post-AI cohort exhibited a greater improvement in the NIHSS score compared to the pre-AI cohort, with a reduction of 4.3 points. While the post-AI cohort demonstrated a higher proportion of good outcomes (mRS 0–1, 26% vs. 40%) at the 3-month follow-up, there was no statistical significance. **Conclusions**: The implementation of Heuron ELVO demonstrated substantial improvements in the timeliness of stroke interventions and patient outcomes. These findings underscore the potential of AI-driven NCCT analysis in enhancing acute stroke workflows and expediting treatments in real-world settings.

## 1. Introduction

Large vessel occlusion (LVO) strokes represent a critical medical emergency that necessitates immediate therapeutic intervention and contributes to significant morbidity and mortality rates in acute ischemic stroke (AIS) [1,2]. The early and accurate diagnosis of LVO strokes is crucial for initiating timely treatment, such as mechanical thrombectomy, to minimize brain damage and improve outcomes [3,4,5].

Traditionally, computed tomography angiography (CTA) or magnetic resonance angiography (MRA) has been the gold standard for diagnosing LVO stroke [6,7]. However, these modalities are not always available, especially in emergency settings. They also have limitations, such as restricted accessibility, delays in imaging, the necessity of expert interpretation, and the risk of contrast-induced complications [8,9].

The incorporation of artificial intelligence (AI) into stroke diagnosis and treatment, particularly in the realm of LVOs, has shown encouraging progress in recent research. Recent studies [10,11,12,13] demonstrate that the integration of AI in stroke management not only aids in accurate and timely diagnosis but also streamlines workflows, offering vital opportunities for enhancing treatment within healthcare systems.

Non-contrast CT (NCCT) is the most used imaging modality to screen for acute stroke, especially intracranial hemorrhage (ICH). Even though several studies have researched LVO on NCCT through the hyperdense artery sign (HAS) as an image-biomarker [14,15,16], it has the limitation of accuracy for use in the clinical environment. Another potential biomarker for screening AIS on NCCT is the Alberta Stroke Program Early Computed Tomography Score (ASPECTS) [17,18,19], which is the quantification-based scoring system for presenting the severity of AIS. However, detecting early stroke signs using NCCT is still challenging, even for experts, due to low contrast and subtle changes in the brain, particularly within the first 3–6 h [20,21,22].

Community or rural hospitals often perform only an NCCT scan initially and subsequently consult a neurologist and/or radiologist to decide whether to perform CTA, CT perfusion or an MR diffusion-weighted image (DWI) in patients with suspected strokes [23]. This practice can lead to significant delays in the diagnosis of LVO and extend the transfer time to a comprehensive stroke center (CSC) for treatment. Furthermore, recent research [24] reported that a substantial number of patients and hospitals experienced time delays between CT and CTA, even in high-performing hospitals in the United States. Therefore, the routine use of CTA for all acute stroke patients may not be the most effective approach, as evaluations using NCCT and clinical examination can identify patients who are likely to benefit from endovascular therapy without unnecessary treatment delays and risks [25].

While most AI tools for LVO diagnosis rely on angiography, there is a growing need for diagnostic AI tools using NCCT, particularly in hospitals without angiography capabilities. The implementation of AI technology for LVO diagnosis via NCCT scans has the potential to revolutionize stroke care by enabling timely treatment in resource-limited environments, providing real-time insights and decision support to healthcare professionals and streamlining the diagnostic process.

In recent years, several studies [23,26,27] have demonstrated the potential of AI tools in detecting LVO on NCCT with promising sensitivity (63.5–83%) and specificity (71–95.1%). However, the impact of these tools on treatment times and clinical outcomes in real-world clinical settings remains unknown, and there is still no study.

To establish scientific evidence regarding how these tools affect various diagnostic processes of acute stroke triage in real-world clinical settings, we evaluated the impact of an AI solution to automatically classify LVO stroke, integrated into the workflow of acute stoke care at a CSC. We hypothesized that this tool would improve treatment times (reduction of door-to-tPA and door-to-EVT times), thereby leading to improvements in stroke benchmarks and clinical outcomes.

## 2. Materials and Methods

### 2.1. Ethic Statement

The study protocols of the current clinical trial were approved by the Institutional Review Board of Gachon University Gil Medical Center (GDIRB2022-037, approval date: 25 January 2022). The ethical standards of the 1964 Declaration of Helsinki and its later amendments were implemented.

### 2.2. Heuron ELVO for Identifying Large Vessel Occlusion (Figure 1)

Heuron ELVO [28] (Heuron StroCare Suit^TM^—ELVO, Heuron Co, Ltd., Seoul, Republic of Korea) is a computer-aided triage and notification software that analyzes NCCT brain images. The device is intended to assist hospital networks and trained stroke experts in workflow triage by flagging findings suggestive of emergent large vessel occlusion (ELVO) in the internal carotid artery (ICA) or middle cerebral artery (MCA) M1 segment on NCCT images. The triage and notification results were derived from this deep learning (DL)-based model to automatically process CT images.

For classifying ELVO cases, three image biomarkers may be identified on NCCT images. The most representative biomarker is the hyperdense artery sign (HAS). HAS is a phenomenon characterized by arterial obstruction due to a thrombus, manifesting as increased density in the middle cerebral artery on NCCT images. Although it is the most representative biomarker of MCA occlusion on NCCT, its presence depends on the slice thickness of the NCCT images. Thus, HAS alone may have low sensitivity in classifying patients in NCCT [14].

A second ELVO biomarker that can be identified in NCCT is eyeball deviation (EBD), which occurs in the direction of the hemisphere affected by the LVO. A study that classified patients with LVO using EBD observed on CT images as a single indicator reported a sensitivity of 71% and a specificity of 77.5% [29].

A third potential biomarker is early ischemic change (EIC) following LVO. EIC represents ischemic changes in brain tissues identified in NCCT images taken at an early stage after stroke onset and is the basis of the ASPECTS [18]. While EIC is not a specific indicator of LVO, its presence may indicate LVO.

NCCT images of 2184 cases (ELVO: 1202 cases, non-ELVO: 982 cases) were used as the learning model for LVO classification. Among these biomarkers, HAS and EIC were identified using CTA and MR diffusion-weighted images by stroke experts. EBD was independently identified by detecting the deviation of the crystalline lens on NCCT. The LVO classification model consists of two major modules. First, the 3D-Convolutional Neural Network (CNN) model identifies the presence of EBD, and HAS, EIC, or old infarction is identified from the 2D-CNN models. Then, a probability value between 0 and 1 is calculated to classify whether an LVO is positive.

After analyzing and detecting ICH and LVO on NCCT, it sends a real-time notification to the stroke care team at the medical facility via mobile/web application, where they can use the image viewer within the application to review the results. The cloud platform facilitates the sharing of diagnostic results and images between the primary stroke center and the CSC for consultation and transfer.

Heuron ELVO received market approval from the Korean Ministry of Food and Drug Safety in 2023 and was designated as an innovative medical technology by the government.

### 2.3. Study Population and Data Collection

**Figure 1:**
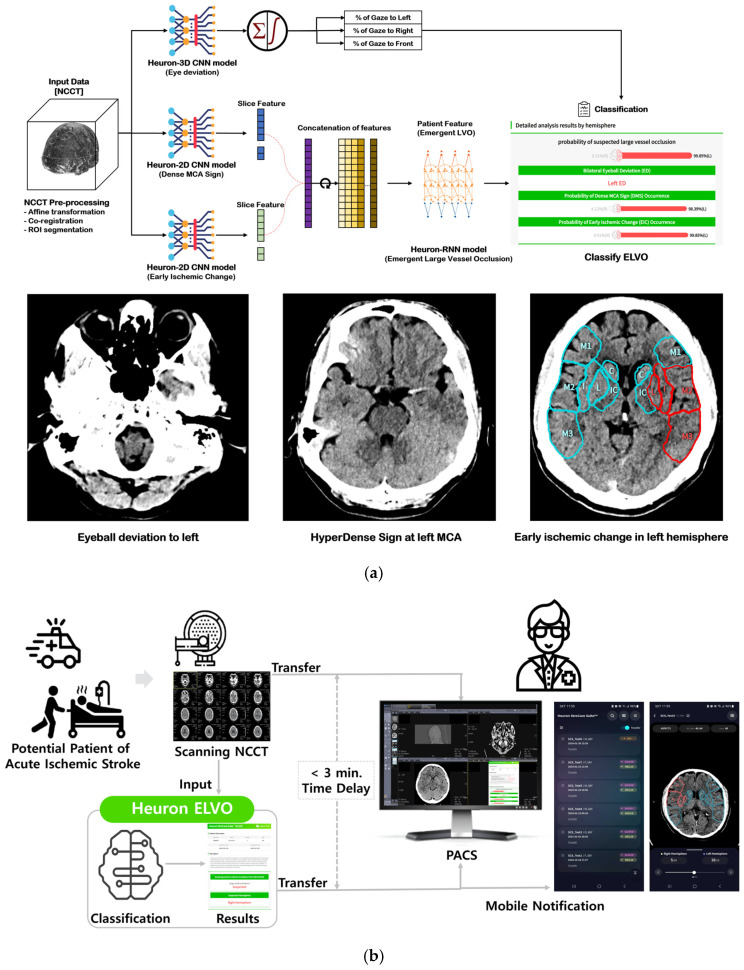
(**a**) The deep-learning model for ELVO classification. Inference flow for classifying patients with large vessel occlusion, and output examples of suspected ELVO cases based on image biomarkers. ELVO: emergent large vessel occlusion; NCCT: non-contrast computed tomography; CNN: convolution neural network; MCA: middle cerebral artery; RNN: recurrent neural network (**b**) A schematic diagram depicting the incorporation of AI-based software (Heuron StroCare Suite^TM^—ELVO v1.0.0.0) into clinical workflow from CT scanner to PACS with mobile application for notification.

This study compared treatment times and clinical outcomes between the pre-AI and post-AI cohorts of AIS patients with LVO who were identified and treated at the emergency and stroke center of Gachon University Gil Medical Center, from 2020 to 2023. We prospectively collected data for the post-AI cohort, consisting of LVO patients who were analyzed using the AI-based LVO triage and notification system, Heuron ELVO, between 1 May 2022 and 31 December 2023. During the prospective test period, when a suspected stroke patient admitted the emergency room and underwent a non-contrast CT test, an AI solution located in the hospital network automatically analyzed the non-contrast CT image. And if the classified result was suspected to be large vessel occlusion, an alarm was sent to the ER medical staff’s mobile app within 3 min as shown in Figure 1. In cases where the analysis result was classified as negative, there was no mobile alert, but the report of the analyzed results in PACS (Picture Archiving and Communication System) could be confirmed. We then matched these patients with a retrospectively collected cohort of LVO patients (pre-AI cohorts) identified using conventional methods between 1 January 2020 and 31 December 2021. Matching characteristics included age (±5 years), sex, time from symptom onset to emergency center arrival (±30 min), and NIHSS score at presentation (±2 points). The critical pathway for stroke patient treatment was the same in both retrospective and prospective periods. However, CT image analysis and result notification were added via Heuron ELVO immediately after non-contrast CT imaging only in the prospective period.

#### 2.3.1. Sample Size Calculation 

The target sample size for prospective cases was determined using G*Power version 3.1.9.7 [30]. We assumed an alpha error of 0.05, a statistical power of 0.8, and an effect size of 0.4, which was estimated using the mean difference between pre-AI and post-AI groups and its standard deviation in prior research on AI-based triage for large vessel occlusion stroke patients [31]. As a result, the sample size of each group was calculated to be at least 41 cases, and retrospective data were collected after the prospectively registered cases met the target number.

#### 2.3.2. Inclusion and Exclusion Criteria

For study inclusion, participants in both cohorts had to meet the following criteria: (1) occlusion at the ICA or M1 segment of MCA; (2) treated with tissue plasminogen activator (tPA), endovascular thrombectomy (EVT), or both; (3) adherence to the protocol of an emergency center visit, NCCT scan, screening, neurologist consultation, and intervention (tPA, EVT, or both); (4) not a transient ischemic attack; and (5) all process from diagnosis to treatment conducted at Gil Medical Center (i.e., no transfers).

Patients with the following criteria were excluded: (1) delays in the decision of tPA or EVT by the patients or the guardian and (2) delays in treatment times due to COVID-19 infection.

**Figure 2:**
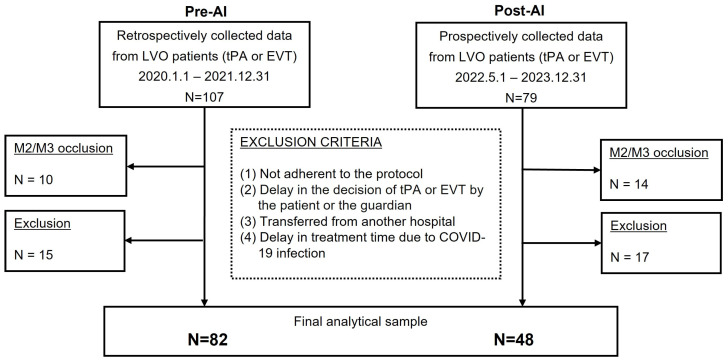
Flowchart of inclusion and exclusion criteria. Individuals in the Pre-AI cohort matched to the Post-AI participants for age ± 5 yrs, sex, time from symptom onset to emergency center arrival ± 30 min, and NIHSS score at presentation ± 2 points. LVO: Large Vessel Occlusion; M: Middle Cerebral Artery; tPA: tissue Plasminogen Activator; EVT: Endovascular Thromectomy.

Considering the inclusion criteria and the gender, age, NIHSS, etc., of the prospectively registered 48 cases, a total of 107 cases were matched retrospectively. After excluding cases that underwent IVT or EVT but did not have MCA M1 occlusion or met the exclusion criteria, 82 cases were matched, and all were enrolled in the analysis. The final analytical sample included 82 participants in the pre-AI cohort and 48 participants in the post-AI cohort (Figure 2).

### 2.4. Outcomes Measurement

The primary outcome was the time difference between arrival at the emergency center and the start of intervention—tPA, EVT, or both. Specifically, door-to-IV tPA and door-to-EVT times were calculated as the time difference in minutes between emergency center arrival and the initiation of tPA or EVT, respectively. For participants who received both treatments, the time differences were calculated separately for each intervention.

The secondary outcomes included (1) the time taken for each step after CT scan and (2) the difference in National Institute of Health Stroke Scale (NIHSS) and modified Rankin Scale (mRS) scores between initial measurements on arrival and those at discharge or at the 3-month follow-up. “CT scan to NR” measured the time taken from CT scan to the neurologist’s examination, while “CT scan-to-IV tPA” and “CT scan to EVT” measured the time from CT scan to the initiation of each respective treatment. Upon arrival at the emergency center, participants were assessed using the NIHSS and mRS. These scores were reassessed at discharge and at the 3-month follow-up, with the differences from the initial scores calculated.

### 2.5. Statistical Analysis

Participants’ sociodemographic and clinical characteristics are summarized as numbers with percentages for categorical variables and as medians with interquartile ranges for continuous variables, after testing for normality. We compared the pre-AI and post-AI cohorts using the Chi-square test, Fisher exact test, or Wilcoxon rank-sum test, as appropriate.

The differences in time and scores in primary and secondary outcomes were calculated as defined and presented as medians with interquartile ranges after testing for normality. Simple comparisons were conducted using the Wilcoxon rank-sum test.

To account for potential confounders, a multiple linear regression model was employed. Covariates were selected a priori and included age (in 10-year increments), sex, the number of comorbidities (including hypertension, diabetes, chronic kidney disease, and atrial fibrillation), NIHSS at presentation, and clot location (ICA or M1 segment of MCA). Using multiple linear regression, the least squares mean with standard errors were estimated for both cohorts, and coefficients for the use of the Heuron LVO were derived in comparison to conventional identification.

All analyses were performed using SAS version 9.4 (SAS Institute Inc., Cary, NC, USA) and R version 4.2.2 (R Foundation for Statistical Computing, Vienna, Austria).

## 3. Results

Table 1 presents the sociodemographic and clinical characteristics of the study participants. The median age of patients was 73 years [interquartile range, 57 to 81] in the pre-AI cohort and 71 years [60 to 79] in the post-AI cohort. The proportion of male patients was over 60% in both groups. Predisposing conditions, including hypertension, diabetes, chronic kidney disease, and atrial fibrillation, were similarly distributed. Initial NIHSS scores were also comparable between the two cohorts, with a median of 13 [10 to 16] in the pre-AI cohort and 13 [9 to 16] in the post-AI cohort. The “door to CT scan”, the interval between emergency center arrival and the CT scan, showed no significant difference. However, there were significant differences in clot locations and types of interventions: Although the occlusion at the M1 segment of the MCA was more prevalent than at the ICA in both cohorts, patients in the post-AI cohort were more likely to have an M1 occlusion (79%) compared to those in the pre-AI cohort (74%). Additionally, the combination of IV tPA and EVT was more frequently used in the pre-AI cohort (54%) than in the post-AI cohort (26%).

The medians of time differences and score differences, along with interquartile ranges, are presented in Table 2. Although the total time from arrival to each intervention decreased after the implementation of Heuron ELVO, these reductions were not statistically significant in a simple comparison using the Wilcoxon rank-sum test. However, when the time interval for each process was examined separately, a significant reduction in CT scan-to-NR times was observed (23 [18 to 37] min in the pre-AI cohort vs. 19 [14 to 23] min in the post-AI cohort). The differences in NIHSS or mRS scores were not statistically significant.

Table 3 summarizes the least squares means with standard errors estimated from a multiple linear regression model. After adjusting for age, sex, the number of comorbidities, NIHSS at presentation, and clot location, the door-to-IV tPA time was shorter in the post-AI cohort (51.9 ± 4.2 min) compared to the pre-AI cohort (60.8 ± 2.8 min), although this difference did not reach statistical significance. In contrast, the door to EVT time showed a significant reduction in the post-AI cohort (138.9 ± 11.9 min vs. 169.1 ± 8.7 min in the pre-AI cohort). Additionally, the post-AI cohort demonstrated significant reductions in the CT scan-to-NR time and CT scan-to-EVT time. Among the score differences in secondary outcomes, only the changes in NIHSS scores from initial presentation to discharge showed a statistically significant improvement in the post-AI cohort.

**Figure 3:**
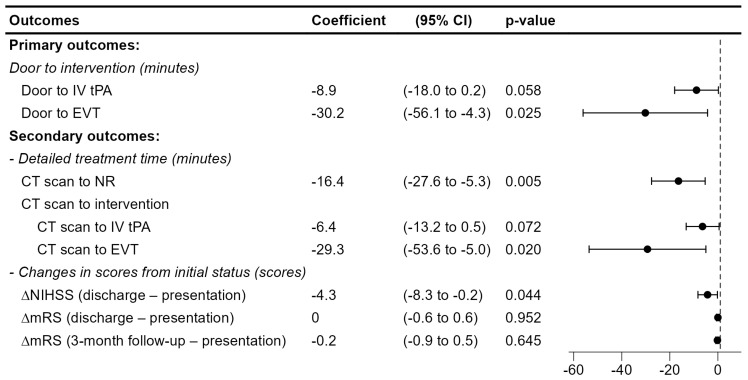
Estimated coefficients of the use of the AI−based triage software using a multivariate linear regression model. Adjustments were made for age (10−year increments), sex, the number of comorbidities, NIHSS at presentation, and clot location. CI, Confidence Intervals, CT: Computed Tomography; NR, Neurologist examination, tPA: tissue Plasminogen Activator; EVT, Endovascular Thrombectomy; NIHSS, National Institutes of Health Stroke Scale; mRS, modified Rankin Scale.

The coefficients of the use of Heuron ELVO were derived from simple and multiple linear regression models and are reported in Table 4. In particular, the coefficients and 95% confidence intervals from the multiple linear regression model are illustrated in Figure 3. Compared to conventional screening in the pre-AI cohort, the implementation of Heuron ELVO in the post-AI cohort was associated with a reduction in the door-to-IV tPA time by 8.9 min (95% CI, −18.0 to −0.2) and a significant reduction in the door-to-EVT time by 30.2 min (−56.1 to −4.3). Moreover, the CT scan-to-NR time and the CT scan-to-EVT time were significantly decreased by 16.4 min (−27.6 to −5.3) and 29.3 min (−53.6 to −5.0), respectively. The change in NIHSS scores from emergency center arrival to discharge showed a significant improvement in the post-AI cohort, with a greater reduction of −4.3 points compared to the pre-AI cohort.

**Figure 4:**
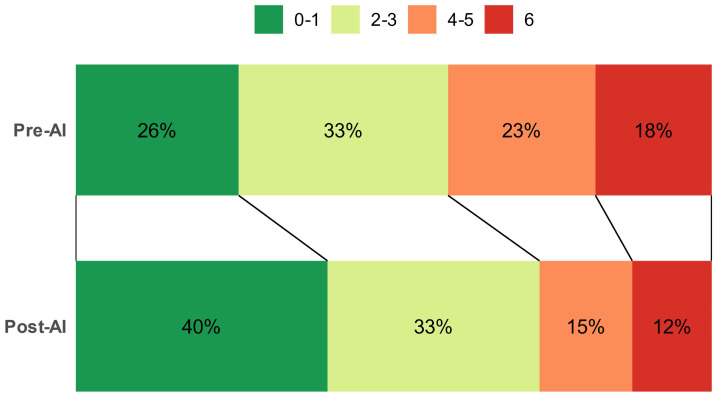
Percentages (%) of mRS at 3−month follow-up.

Although a higher proportion of patients in the post-AI cohort reported lower mRS scores at the 3-month follow-up compared to those in the pre-AI cohort (Figure 4), the adjusted coefficients indicated that the reduction in mRS scores was not substantial.

## 4. Discussion

Our study evaluated the impact of AI solutions for the triage and notification of emergent LVO stroke on treatment times and clinical outcomes in AIS patients presenting with LVO. Our results suggest that the use of AI technology in diagnosing LVO strokes from NCCT scans has shown promising results in reducing treatment times and improving patient outcomes. We found that the implementation of the AI solution, the Heuron ELVO, resulted in a 30.2 min reduction in the door-to-EVT time, a 16.4 min reduction in the CT-to-NR time, and a 29.3 min reduction in the CT-to-EVT time, as determined by multiple linear regression analyses compared to the pre-AI cohort. Additionally, we observed an improvement in NIHSS scores in the post-AI cohort compared with the pre-AI cohort. While door-to-CT times did not show a significant difference, the significant reduction of CT-to-EVT times suggests that the integration of Heuron ELVO has a substantial impact on the most time-sensitive part of the stroke care pathway.

The stroke community should develop practical, efficient, and affordable protocols to triage patients for endovascular therapy that can be applied expeditiously and on a large scale in hospitals [25]. Nowadays, CTA has become an essential component of acute stroke imaging for advanced treatment selection, such as the use of tPA and mechanical thrombectomy in AIS [6,32]. In recent years, there has been a surge in the development of AI-powered automated software capable of detecting LVOs using CTA, which is being used commercially. Among these, Viz LVO (Viz ai, Inc., San Fransisco, VA, USA) and RAPID CTA (SchemaView, Menlo Park, CA, USA) have emerged as leading platforms, offering rapid and accurate LVO detection to aid in timely treatment. These AI software platforms have been shown to streamline stroke workflows and reduce door-to-thrombectomy times [33,34,35,36,37,38,39].

However, there are practical challenges in the routine use of CTA, such as the lack of CTA capabilities in many smaller and rural hospitals, the possibility of treatment delays due to the time required to perform or interpret a CTA, and the risk of complications from contrast exposure. According to recent research [24] conducted on 23,925 patients in 717 high-performing hospitals in the United States, a substantial number of patients (>15 min: 18%) and hospitals (>15 min: 17%) experienced time delays between CT and CTA in even high-performing hospitals. Therefore, routine use of CTA for all acute stroke patients may not be the most effective approach, as the evaluation of NCCT and clinical examination can identify patients who are likely to benefit from endovascular therapy without unnecessary treatment delays and risks [25].

Although NCCT offers a valuable alternative with several advantages compared to CTA, it has relatively lower sensitivity in detecting EIC and may require interpretation by stroke specialists. Recent advancements in AI technology enable NCCT to effectively identify LVO strokes that are likely to benefit from thrombectomy with comparable accuracy to CTA, thereby streamlining the treatment process and reducing unnecessary delays in treatment. While recent studies have demonstrated the potential of AI tools to accurately predict LVO using NCCT scans [23,26,27], there was no study that explored its integration into existing stroke management workflows. Therefore, we performed this study because the potential for AI tools to reduce diagnostic times and improve patient outcomes should be a key area of investigation.

The diagnosis of LVO on NCCT using an AI solution has mainly focused on predicting clot signs or early ischemic change (EIC). ASPECTS has been extensively utilized globally to assess the extent of ischemic changes on NCCT in patients with AIS [17,18,19]. Despite its simplicity, identifying early stroke signs remains challenging even for experts due to low contrast and subtle brain changes. Consequently, the AI-based automated ASPECTS tool can support physicians’ interpretations and enhance accuracy.

In recent years, the evidence that automated scoring methods of ASPECTS based on machine learning are comparable with expert reading of ASPECTS is accumulating. Two commercially available software programs exist for automated evaluation of ASPECTS: the e-ASPECTS software (Brainomix, Oxford, UK) [40] and RAPID ASPECTS program (iSchemaView, Menlo Park, CA, USA) [41].

e-ASPECTS has been shown to be non-inferior or even superior in diagnostic accuracy compared with stroke physicians [42] or neuroradiologists [43]. RAPID ASPECTS show higher agreement (intraclass correlation coefficient (ICC), 0.55) with DWI ASPECTS than clinician scores (ICC, 0.29) [44], suggesting its clinical relevance for patient selection in thrombectomy trials. Another automated scoring method of ASPECTS [45] based on feature engineering and random forest learning demonstrated a strong agreement with expert-read DWI ASPECTS, achieving an ICC of 0.76 and a mean difference of 0.3 for total ASPECTS. For individual ASPECTS regions, the method yielded a sensitivity of 66.2%, a specificity of 91.8%, and an area under the curve (AUC) of 0.79.

Heuron StroCare Suite^TM^ also included a DL-based automated scoring system of ASPECTS (Heuron ASPECTS) as a component of the analyzing tool. Our current trial [46] results showed that Heuron ASPECTS reliably measures the ASPECTS for use in clinical practice. Heuron ASPECTS demonstrated reliable performance with a mean difference of 0.03 in scoring compared to expert consensus, achieving a sensitivity of 62.78% and specificity of 96.63% for detecting EIC of 10 ASPECTS regions. Furthermore, in a dichotomized analysis (ASPECTS > 4 vs. ≤4), sensitivity was 94.01% and specificity was 61.90%. This performance is comparable to that of other automated ASPECTS software tools like e-ASPECTS [47], Frontier ASPECTS [47], and RAPID ASPECTS [44], which were also found to have reasonable performance in a reference study.

HAS [14,15,16] is the most representative indicator for LVO identification on NCCT. Several studies reported that the automated detection of HAS by an AI-driven software application on NCCT images from patients with AIS due to LVO showed similar performance as that of trained physicians. Weyland et al. [48] reported that AI-driven Brainomix demonstrated a sensitivity of 77% and specificity of 87% for detecting HAS, comparable to trained neuroradiologists who had sensitivities of 80% and 93% and specificity of 71% and 93%, respectively. Another study [26] with MethinksLVO demonstrated high accuracy in predicting LVO on NCCT, with an AUC of 0.87 (sensitivity of 83%, specificity of 71%), which improved to 0.91 (sensitivity of 83%, specificity of 85%) when additional clinical data (NIHSS) were added to the model. You et al. [49] established an automated Hierarchy Evaluation System using machine learning for acute LVO stroke, combining structured clinical data with non-structured NCCT imaging data. The model demonstrated superior performance (sensitivity of 95.3%, specificity of 68.4%) compared to previous approaches, potentially enhancing prehospital triage systems for AIS. HAS indicates a high likelihood of arterial obstruction, but the presence or absence of HAS alone is insufficient to diagnose LVO stroke [14].

Recently, there have been several AI-based models using both HAS and ASPECTS in NCCT. Yedavalli et al. [23] evaluated the RAPID NCCT Stroke platform, which uses neural networks and automated segmentation techniques based on predefined thresholds for the identification of ICH, HAS, and ASPECTS. They demonstrated superior sensitivity performance (63.5%) to general radiologists (40.9%) and neuroradiologists (43.6%) for detecting LVO from NCCT. Heuron ELVO additionally evaluates EBD, which is a distinguishing feature from other AI tools. EBD observed on CT scans is associated with LVO and can be used to predict LVO in AIS. One study [29] reported radiological EBD on CT scans demonstrated a sensitivity of 71% and a specificity of 77.5% in predicting LVO, metrics comparable to those of the NIHSS, which exhibited a sensitivity of 78.5%, and a specificity of 76.1%. Heuron ELVO uses a DL algorithm that is based on the detection of HAS, EIC, and imaging-based EBD because no single biomarker is sufficient on its own for predicting LVO.

In our previous study [50,51] with 477 patients who were suspected of having AIS, we demonstrated that Heuron ELVO-assisted LVO diagnosis in NCCT significantly improved diagnostic accuracy. Based on consensus results of stroke expert readers, Heuron ELVO-assisted evaluations resulted in higher sensitivity (75.9% vs. 92.0%, *p* = 0.009) and specificity (83.0% vs. 92.6%, *p* < 0.0001) than the unassisted evaluations. Further, when the interrater reliability of agreement among readers was analyzed at each stage, the kappa value was 0.27 (95% CI: 0.26–0.28) for the unassisted evaluation, which increased to 0.75 (95% CI: 0.74–0.76) for Heuron ELVO-assisted evaluation.

The integration of Heuron ELVO in a real-world setting revealed the reduction of door-to-treatment times, particularly CT-to-NR and CT-to-EVT times. While there have been many studies [33,34,35,36,37,38,39] on the clinical effectiveness of AI-based LVO detection tools using CTA, to the best of our knowledge, this is the first study that evaluated the impact of AI-based LVO detection and notification software using NCCT on acute stroke workflows and clinical outcomes in real-world settings.

Accurate prediction of LVO in NCCT by AI tools might spare further neuroimaging exams like CTA in selected patients, and the interpretation of NCCT by stroke experts for deciding whether to perform CTA is not needed. It can support radiologists, neurologists, and interventionalists in assessing stroke findings from an NCCT and a simple neurological exam; thereby, it may improve accuracy in the diagnosis of LVO stroke. In addition, real-time notifications to stroke specialists can provide them with quick access to the result of AI analysis on NCCT scans. This streamlined approach could lead to significant reductions in time and resources spent on unnecessary imaging. Furthermore, a real-time notification to the stroke care team via mobile/web application enables rapid preparation and intervention in a complex hospital environment. This innovative integration of AI technology not only enhances communication among stroke teams but also ensures that critical interventions are initiated without delay, ultimately leading to better management of AIS patients and favorable outcomes.

The most important point is that in resource-limited small and rural hospitals, the implementation of AI-driven LVO detection on NCCT enables the rapid transfer of LVO stroke patients requiring thrombectomy to CSCs without unnecessary delay, while also reducing unnecessary transfer. This ongoing evolution in stroke management highlights the importance of leveraging technology to optimize patient care and outcomes, making it an essential component of modern healthcare strategies.

In evaluating the efficacy of AI software in stroke care, it is necessary to assess not only its clinical utility but also its economic benefits. Although there is still no direct evidence that the application of AI software for stroke care provides greater economic benefits than conventional clinical practice, several studies on cost-effectiveness analysis have reported that the improvement in reperfusion rates and the reduction in time to thrombectomy resulted in significant healthcare and societal cost savings [52,53,54]. Furthermore, the use of AI tools to assist with mechanical thrombectomy has the potential to reduce costs by decreasing the misdiagnosis of LVO stroke [55]. A comprehensive evaluation of the cost-benefit analysis of AI software integration in clinical settings is warranted.

While current studies show high diagnostic performance, the implementation of AI in clinical decision making has potential challenges, such as the variability in medical interpretations and observations, which are often overlooked in analyses [56]. The performance of AI models can vary across different populations and CT scanners, necessitating careful optimization and training to account for demographic diversity across various healthcare settings [57]. Therefore, further multicenter studies with a larger cohort are warranted to validate the clinical utility of Heuron ELVO and refine it in various healthcare settings. Furthermore, many challenges, such as data quality, algorithmic transparency, and ethical ramifications, persist [58]. Further research is crucial to address these technical, ethical, and regulatory hurdles.

Our study has several limitations. First, the non-randomized, non-blinded design, and single-center nature of the study may have introduced selection bias, limiting the generalizability of our findings to other healthcare settings. Additionally, attention bias should be considered, as clinicians may have been more aware of and responsive to time-sensitive processes, potentially affecting the improvements. To mitigate this limitation, we employed matching to balance baseline characteristics, adjusted for potential confounders, and included a retrospectively collected control group of stroke patients. Nonetheless, the findings should be interpreted with caution. Second, our study primarily focused on proximal MCA territory strokes, which limits the generalizability of the model to other stroke locations and mechanisms. The model’s performance may vary across different CT scanner platforms, acquisition protocols, diverse patient populations and varying clinical settings, which requires further validation studies. Third, although we excluded patients with COVID-19 infection from the study populations, the study period coincided with the COVID-19 pandemic, which may have influenced our results. To address these limitations, we are currently conducting multicenter clinical trials to confirm these findings in a larger and diverse population.

## 5. Conclusions

The implementation of the AI software based on NCCT, Heuron ELVO, in acute stroke care was associated with a significant reduction of treatment times, including CT-to-NR times, CT-to-EVT times, and door-to-EVT times, as well as improved clinical outcomes. This suggests that AI-driven NCCT analysis for LVO stroke can contribute to streamlining LVO stroke workflows and expediting treatment in real-world settings.

## Figures and Tables

**Table 1:** Baseline characteristics of pre-AI and post-AI cohorts.

Variables	Pre-AI (n = 82)	Post-AI (n = 48)	*p*-Value
Age (years)	73 [57 to 81]	71 [60 to 79]	0.773
Sex, Male (%)	49 (60)	32 (67)	0.362
Past medical history (%)			
Hypertension	52 (63)	28 (58)	0.164
Diabetes	27 (33)	13 (27)	0.101
Chronic kidney disease	2 (2)	1 (2)	1.000
Atrial fibrillation	26 (32)	15 (31)	0.989
NIHSS at presentation (scores)	13 [10 to 16]	13 [9 to 16]	0.973
mRS at presentation (scores)	5 [4 to 5]	4 [4 to 5]	<0.001
Door to CT scan (minutes)	17 [12 to 29]	18 [13 to 21]	0.434
Clot location (%)			0.029
Internal carotid artery	21 (26)	10 (21)	
M1 middle cerebral artery	61 (74)	38 (79)	
Intervention (%)			0.012
IV tPA alone	12 (15)	13 (27)	
EVT alone	26 (32)	21 (44)	
IV tPA + EVT	44 (54)	14 (29)	

Baseline characteristics are presented as medians with interquartile ranges for continuous variables or as numbers with percentages for categorical variables. The comparisons were conducted between two groups using the Wilcoxon rank-sum test, Chi-square test, or Fisher exact test, as appropriate. tPA, tissue Plasminogen Activator; EVT, Endovascular Thrombectomy; NIHSS, National Institutes of Health Stroke Scale; mRS, modified Rankin Scale.

**Table 2:** Comparison of primary and secondary outcomes between pre-AI and post-AI cohorts in a univariate analysis.

Variables	Pre-AI (n = 82)	Post-AI (n = 48)	*p*-Value
**Primary outcomes:**			
Door to intervention (minutes)			
Door to IV tPA	56 [47 to 60]	52 [43 to 57]	0.072
Door to EVT	154 [129 to 177]	146 [127 to 164]	0.137
**Secondary outcomes:**			
Detailed treatment time (minutes)			
CT scan to NR	23 [18 to 37]	19 [14 to 23]	<0.001
CT scan to intervention			
CT scan to IV tPA	37 [31 to 47]	33 [26 to 42]	0.104
CT scan to EVT	132 [112 to 160]	126 [111 to 146]	0.191
Changes in scores from initial status (score)			
∆NIHSS (discharge—presentation)	−5 [−9 to −1]	−7 [−10 to −4]	0.106
∆mRS (discharge—presentation)	−1 [−2 to 0]	−1 [−2 to 0]	0.721
∆mRS (3-month follow-up—presentation)	−2 [−3 to 0]	−2 [−3 to −1]	0.462

Results are presented as medians with interquartile ranges after testing for normality and were compared using the Wilcoxon rank-sum test. CT, Computed Tomography; NR, Neurologist examination; tPA, tissue Plasminogen Activator; EVT, Endovascular Thrombectomy; NIHSS, National Institutes of Health Stroke Scale; mRS, modified Rankin Scale.

**Table 3:** Comparison of primary and secondary outcomes between pre-AI and post-AI cohorts using a multiple linear regression model.

Variables	Pre-AI (n = 82)	Post-AI (n = 48)	*p*-Value
**Primary outcomes:**			
Door to intervention (minutes)			
Door to IV tPA	60.8 (±2.8)	51.9 (±4.2)	0.058
Door to EVT	169.1 (±8.7)	138.9 (±11.9)	0.025
**Secondary outcomes:**			
Detailed treatment time (minutes)			
CT scan to NR	31.2 (±3.9)	14.8 (±5.1)	0.005
CT scan to intervention			
CT scan to IV tPA	40.1 (±2.1)	33.8 (±3.2)	0.072
CT scan to EVT	147.8 (±8.1)	118.5 (±11.2)	0.020
Changes in scores from initial status (score)			
∆NIHSS (discharge—presentation)	−1.4 (±1.5)	−5.7 (±1.9)	0.044
∆mRS (discharge—presentation)	−1.2 (±0.2)	−1.1 (±0.3)	0.952
∆mRS (3-month follow-up—presentation)	−1.6 (±0.2)	−1.7 (±0.3)	0.645

Results were estimated using a multiple linear regression model and are presented as least squares means with standard errors. The multiple linear regression model was adjusted for age (in 10-year increments), sex, the number of comorbidities, NIHSS at presentation, and clot location. CT, Computed Tomography; NR, Neurologist examination; tPA, tissue Plasminogen Activator; EVT, Endovascular Thrombectomy; NIHSS, National Institutes of Health Stroke Scale; mRS, modified Rankin Scale.

**Table 4:** Associations between the use of the AI-based triage software and primary and secondary outcomes.

Variables	Simple Regression		Multiple Regression	
	Coefficient (95% CI)	*p*-Value	Coefficient (95% CI)	*p*-Value
**Primary outcomes:**				
Door to intervention (minutes)				
Door to IV tPA	−9.2 (−17.8 to −0.5)	0.041	−8.9 (−18.0 to 0.2)	0.058
Door to EVT	−29.6 (−55.4 to −3.9)	0.026	−30.2 (−56.1 to −4.3)	0.025
**Secondary outcomes:**				
Detailed treatment time (minutes)				
CT scan to NR	−15.5 (−26.6 to −4.5)	0.007	−16.4 (−27.6 to −5.3)	0.005
CT scan to intervention				
CT scan to IV tPA	−6.1 (−12.6 to 0.5)	0.074	−6.4 (−13.2 to 0.5)	0.072
CT scan to EVT	−28.1 (−52.5 to −3.7)	0.026	−29.3 (−53.6 to −5.0)	0.020
Changes in scores from initial status (score)				
∆NIHSS (discharge—presentation)	−4.8 (−9 to −0.6)	0.027	−4.3 (−8.3 to −0.2)	0.044
∆mRS (discharge—presentation)	0.0 (−0.6 to 0.6)	0.916	0.0 (−0.6 to 0.6)	0.952
∆mRS (3-month follow-up—presentation)	−0.2 (−0.9 to 0.5)	0.513	−0.2 (−0.9 to 0.5)	0.645

The coefficients of the AI-based stroke detection system were estimated in a multiple linear regression model, compared to non-use. The model was adjusted for age (in 10-year increments), sex, the number of comorbidities, NIHSS at presentation, and clot location. CT, Computed Tomography; NR, Neurologist examination; tPA, tissue Plasminogen Activator; EVT, Endovascular Thrombectomy; NIHSS, National Institutes of Health Stroke Scale; mRS, modified Rankin Scale.

## Data Availability

The datasets generated and analyzed during the current study are not publicly available since they contain potentially identificatory information for each patient; however, they are available from the corresponding author upon reasonable request.
